# Nomogram for predicting axillary pathologic complete response after neoadjuvant systemic therapy in HER2 positive and triple negative breast cancer

**DOI:** 10.7150/jca.118908

**Published:** 2025-09-22

**Authors:** Zhendong Shi, Hanyan Zhu, Xiaoxing Bian, Xiaomin Qian, Jie Meng, Jin Zhang

**Affiliations:** 1 The Third Department of Breast Cancer, Tianjin Medical University Cancer Institute and Hospital, National Clinical Research Center for Cancer, Tianjin, China.; 2 Key Laboratory of Cancer Prevention and Therapy, Tianjin Medical University Cancer Institute and Hospital, Tianjin, China.; 3 Tianjin's Clinical Research Center for Cancer, Tianjin Medical University Cancer Institute and Hospital, Tianjin, China.; 4 Key Laboratory of Breast Cancer Prevention and Therapy, Tianjin Medical University, Ministry of Education, Tianjin, China.; 5 Department of Medical Laboratory, School of Medical Technology, Tianjin Medical University, Tianjin, China.

**Keywords:** Axillary lymph node metastasis, Neoadjuvant therapy, HER2 positive breast cancer, Triple-negative breast cancer, Predictive nomogram

## Abstract

**Purpose:** With the continuous improvement in the efficacy of neoadjuvant therapy (NAT), a significant proportion of breast cancer patients initially diagnosed with pathologically confirmed axillary lymph node metastasis (pN+) may achieve ypN0 status (no residual nodal metastasis) following NAT. This study aims to develop a predictive model for estimating the probability of achieving ypN0 status after NAT, thereby assisting surgeons in making optimal decisions regarding axillary management strategies.

**Methods:** This retrospective study enrolled 671 patients diagnosed with pN+ at Tianjin Medical University Cancer Institute and Hospital between December 2018 and December 2022, all of whom completed NAT followed by surgical intervention. The cohort comprised 428 HER2-positive and 243 TNBC patients. Clinicopathological and ultrasound imaging data were systematically collected. Patients were stratified into training and validation sets at a 7:3 ratio based on admission dates. Univariate analysis was initially performed on the training set to identify potential factors associated with achieving ypN0 status post-NAT. Variables demonstrating statistical significance were subsequently incorporated into a multivariate logistic regression analysis to determine independent predictors. A predictive nomogram was then constructed using these independent factors via R software for visual interpretation of ypN0 probability. The predictive performance of the model was ultimately evaluated by generating receiver operating characteristic (ROC) curves to assess discriminative ability and calibration curves to quantify prediction accuracy, with further validation performed using the independent validation cohort.

**Results:** In HER2 positive breast cancer patients, those exhibiting histological grade III, HER2 IHC 3+ expression, absence of lymphovascular invasion, clinical N1 stage, prominent and hypervascular tumor CDFI signal pre-NAT, and achievement of breast pathological complete response (bpCR) following NAT were significantly more likely to achieve ypN0 status. Conversely, among TNBC patients, independent predictors of post-NAT ypN0 achievement included histological grade III, taxane-platinum combination regimens, bpCR, dot-linear signals in axillary lymph nodes on post-NAT ultrasound, and minimal transverse diameter of node on final post-NAT ultrasound evaluation.

**Conclusions:** This study established distinct predictive models for HER2-positive and TNBC cohorts with initial pN+ status to estimate the probability of achieving ypN0 following NAT. Both models demonstrated robust predictive performance through rigorous validation, providing clinicians with quantitative tools to optimize axillary management strategies and facilitate precision-based individualized treatment planning.

## Introduction

Breast cancer has emerged as the most frequently diagnosed malignancy among women under 40 years of age and the leading cause of cancer-related mortality in females globally, accounting for 15% of female cancer-related deaths 1-2. In China, approximately half of breast cancer patients are diagnosed before the age of 50 and during premenopausal status, with these cases often exhibiting more aggressive subtypes such as triple-negative breast cancer (TNBC) 3. The rising incidence of breast cancer among younger women underscores the critical need to improve both survival rates and post-treatment quality of life, highlighting its profound clinical and public health significance.

Neoadjuvant therapy (NAT), encompassing chemotherapy, targeted therapy, and endocrine therapy, primarily aims to reduce primary breast tumor volume and downstage metastatic axillary lymph nodes. This enables surgical resection in initially inoperable patients and expands breast-conserving eligibility for those previously deemed unsuitable for such procedures 4. Pathological complete response (pCR) has been established as a validated surrogate endpoint in neoadjuvant clinical trials and a robust biomarker of NAT efficacy 5. Numerous studies demonstrate that TNBC and HER2-positive subtypes exhibit heightened sensitivity to NAT 6, 7. Furthermore, patients with clinically node-positive disease at initial diagnosis achieve pCR status more frequently within these two subtypes following NAT. Accordingly, these two patient cohorts were enrolled in this study. Beyond histopathological parameters, imaging modalities provide real-time and actionable insights into therapeutic response. Breast ultrasonography, characterized by its non-invasive nature, cost-effectiveness, and procedural accessibility, serves as a critical monitoring tool 8. Emerging evidence from ultrasound radiomics research further supports its predictive utility for pCR achievement post-NAT across both early-stage and advanced breast cancer cohorts 9, 10.

Axillary lymph node dissection (ALND) and sentinel lymph node biopsy (SLNB) represent two standardized surgical approaches for axillary staging in contemporary breast cancer management. While ALND remains the most accurate modality for axillary staging, it is associated with substantial morbidity, including hematoma formation, chronic pain, restricted shoulder mobility, and axillary web syndrome, all of which adversely impact postoperative recovery and long-term quality of life 11, 12. In contrast, SLNB offers reduced surgical trauma and significantly lower complication rates compared to ALND 13. The 8-year follow-up data from the phase III randomized NSABP B-32 trial demonstrated that SLNB could safely replace ALND in clinically node-negative (cN0) patients, achieving comparable staging accuracy and equivalent oncological outcomes 14.

With advancements in pharmacological agents and therapeutic strategies, the efficacy of NAT has significantly improved. Studies report that the probability of clinically node-positive (cN+) breast cancer patients achieving clinically node-negative status post-NAT varies across cohorts, reaching up to 91% in select populations 15-17. A study from the European Institute of Oncology analyzed 222 patients with cT1-3, cN1-2 breast cancer who achieved clinically node-negative status following NAT and underwent SLNB. The 10-year follow-up data confirmed favorable overall survival in this cohort, particularly among HER2-positive and triple-negative subtypes 16. The potential omission of ALND in patients with pretreatment-confirmed axillary lymph node metastasis, especially those with initial N1-3 disease, has emerged as a critical clinical question. This paradigm shift toward less invasive approaches aims to mitigate overtreatment-related morbidity while maintaining oncological safety. However, the feasibility and long-term safety of ALND de-escalation in this population require further validation through large-scale prospective trials and standardized response assessment protocols.

The present study investigated clinicopathological and imaging characteristics of HER2-positive and TNBC patients with pathologically confirmed axillary lymph node metastasis at initial diagnosis to identify predictors of achieving ypN0 status following NAT. A predictive model was subsequently developed to guide surgical decision-making in axillary management, aiming to optimize individualized treatment strategies while minimizing overtreatment-related morbidity.

## Materials and Methods

### Patient population and study design

This study enrolled breast cancer patients diagnosed with HER2-positive or TNBC accompanied by pN+, confirmed via core needle biopsy, who completed NAT and subsequent surgical intervention at Tianjin Medical University Cancer Institute and Hospital between December 2018 and December 2022. The final cohort comprised 671 patients, including 428 HER2-positive and 243 TNBC cases. Clinicopathological characteristics, pre-NAT and post-NAT ultrasound imaging data, and postoperative histopathological findings were systematically collected for analysis.

**Inclusion criteria**: (1) Female patients diagnosed with HER2-positive or triple-negative breast cancer subtypes; (2) Pathologically confirmed pN+ via pre-NAT core needle biopsy; (3) Completion of NAT followed by surgical intervention with ALND at our institution; (4) Availability of complete clinicopathological and treatment response documentation.

**Exclusion criteria:** (1) Patients with inflammatory breast cancer, occult breast cancer, or bilateral breast cancer; (2) Prior receipt of cytotoxic therapy, localized radiotherapy, endocrine therapy, immunotherapy, or other anticancer treatments before NAT; (3) Partial or complete resection of the primary tumor prior to NAT initiation; (4) Presence of distant metastasis at initial diagnosis; (5) Concomitant primary malignancies other than breast cancer; (6) Severe systemic comorbidities (e.g., uncompensated cardiac, hepatic, or renal dysfunction).

### Clinicopathological characteristics and efficacy assessments

The clinical baseline characteristics of enrolled patients were retrospectively collected, including age, menstrual status, gravidity, tumor location in the breast, clinical staging of the tumor, NAT regimen, NAT cycles, and surgical approach for breast cancer. Clinical stage was performed according to the 8th edition of the American Joint Commission Cancer (AJCC) tumor-node-metastasis (TNM) staging system. Molecular subtypes were determined based on immunohistochemical staining results of the primary tumor before NAT. ER/PR expression status: Positive expression was defined when nuclear-stained tumor cells accounted for ≥1% of total tumor cells, otherwise classified as negative. HER2 expression status: positive expression was defined IHC 3+ or IHC 2+/FISH-positive (with HER2 gene amplification). Ki67 was defined as low when the percentage of stained cells was < 20% and high when ≥ 20%. TILs (tumor-infiltrating lymphocytes) level: TILs were categorized using a 10% cut-off value, with <10% defined as low TILs infiltration and ≥10% defined as moderate-to-high TILs infiltration. TNBC was defined as negative expression of ER, PR, and HER2, with any level of Ki-67 expression. HER2-positive breast cancer was defined as HER2-positive expression, regardless of ER/PR status (positive or negative).

The Response Evaluation Criteria in Solid Tumors version 1.1 (RECIST 1.1) were applied to assess the overall clinical efficacy of NAT in patients, with outcomes categorized into four types as follows:

**Complete Response (CR):** Complete disappearance of all target lesions;

**Partial Response (PR):** A reduction of ≥30% in the sum of the diameters of target lesions compared to baseline;

**Progressive Disease (PD):** An increase of ≥20% in the sum of the diameters of target lesions relative to baseline or the appearance of one or more new lesions;

**Stable Disease (SD):** Changes in the sum of target lesion diameters that do not meet the thresholds for PR or PD 18.

Assessment criteria for pCR after NAT included ypTis/0ypN0 (defined as absence of invasive cancer or ductal carcinoma *in situ* in the breast and axillary lymph nodes).

### Treatment protocols

All patients received at least 4 cycles of standard NAT before surgery. TNBC patients predominantly received 4-6 cycles of combination chemotherapy, primarily consisting of taxane-based regimens combined with anthracyclines (TA) or platinum-based agents (TP). Patients with HER2-positive breast cancer universally received targeted therapy with either trastuzumab monotherapy or dual HER2 blockade using trastuzumab plus pertuzumab. Chemotherapy regimens for HER2-positive disease comprised: 4-6 cycles of single-agent taxane, taxane plus carboplatin combinations, or anthracycline-cyclophosphamide followed by sequential taxane therapy (totaling 8 cycles). RECIST 1.1 was used for the evaluation of the clinical response. The modified radical mastectomy or breast-conserving surgery for breast cancer was performed between 21 to 35 days after the patient completed the final NAT treatment, based on the current therapeutic outcomes and the patient's personal preference. All patients also received axillary lymph node dissection.

### Ultrasound imaging data

Collect the baseline ultrasound imaging data of enrolled patients prior to the first NAT and post-NAT ultrasound imaging data before surgery after the final NAT session. Specific parameters include: maximum tumor diameter, peritumoral echo characteristics, posterior echo features, tumor Color Doppler Flow Imaging (CDFI) signals, minimal diameter of axillary lymph nodes, CDFI signals in axillary lymph nodes, and presence or absence of hyperechoic medullary signals in axillary lymph nodes. Through comparative analysis of these parameters, disease progression data after NAT treatment will be obtained, including:

Primary tumor response rate: (pre-NAT maximum tumor diameter - post-NAT maximum tumor diameter) / pre-NAT maximum tumor diameter

Axillary lymph node response rate: (pre-NAT minimal lymph node diameter - post-NAT minimal lymph node diameter) / pre-NAT minimal lymph node diameter

### Statistical analysis

Univariate analysis was performed using the Chi-square test and Fisher's exact test to identify factors associated with achieving ypN0 status after NAT. Significant factors from this analysis were subsequently incorporated into a multivariate logistic regression model to determine independent predictive factors. All statistical analyses were conducted using SPSS 25.0, with a significance threshold of *p*<0.05. Based on the multivariate results, a predictive model was constructed and visualized as a nomogram using R software (version 4.4.2). The model's discriminative ability was evaluated by generating a ROC curve and calculating the area under the curve (AUC). Calibration curves were plotted to assess the model's accuracy, and the model was further validated using an independent validation cohort.

## Results

### Clinicopathological and ultrasonographic characteristics of the baseline training and validation sets

**Figure 1:**
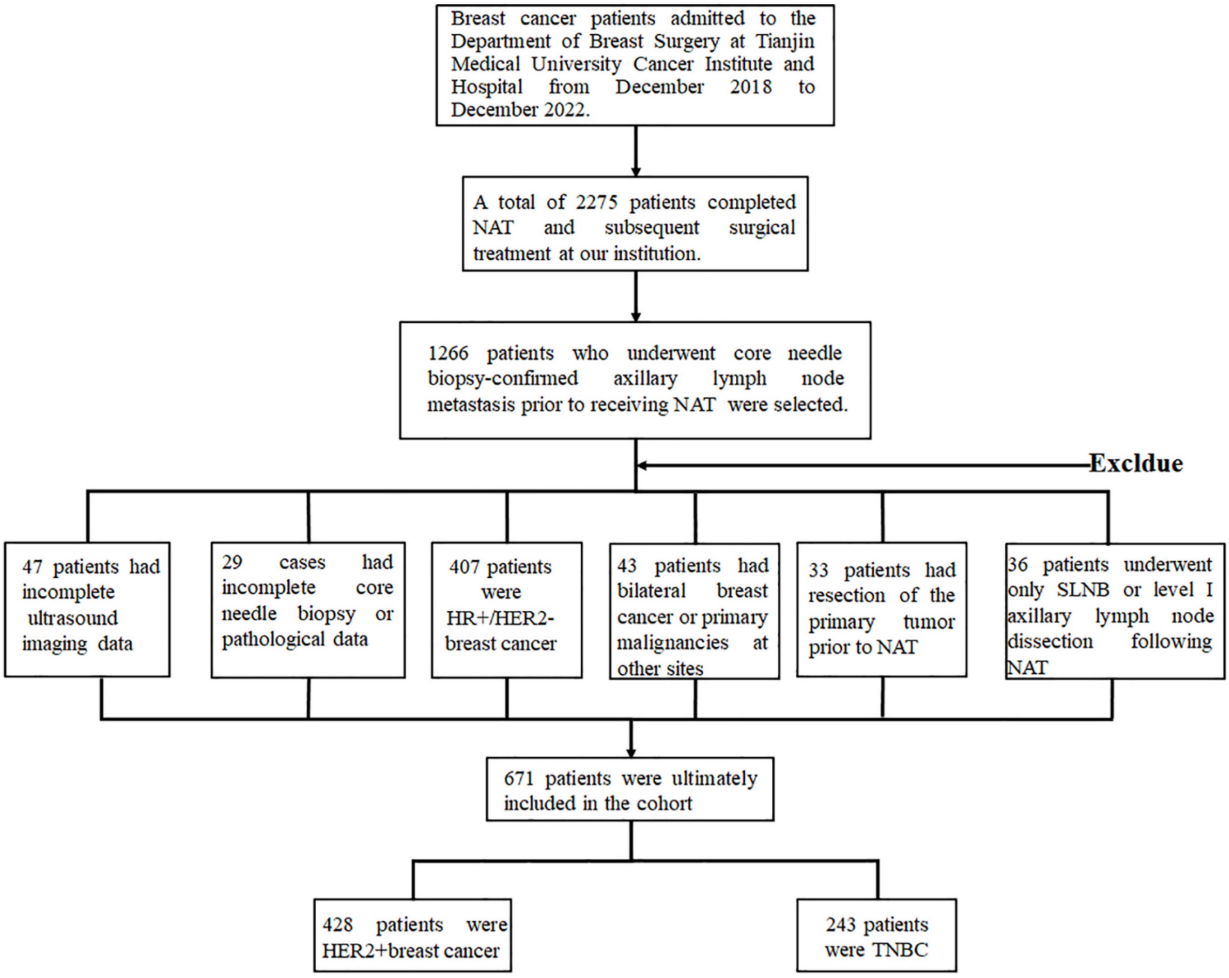
Study flowchart of HER2- positive and TNBC patients enrollment process.

This study retrospectively enrolled patients with pathologically confirmed axillary lymph node metastasis who underwent NAT followed by surgical treatment at our institution. Exclusion criteria were as follows: incomplete imaging data (n=47), incomplete clinicopathological records (n=29), HR-positive/HER2-negative subtype (n=407), bilateral breast cancer or concurrent primary malignancies at other sites (n=43), prior excision of the primary lesion before NAT (n=33), and post-NAT axillary intervention limited to sentinel lymph node biopsy or level I lymph node dissection (n=36). A total of 671 patients were ultimately included in the analysis, comprising 428 HER2-positive and 243 triple-negative breast cancer cases (Figure 1).

Patients were stratified into training and validation cohorts at a 7:3 ratio based on their date of admission, resulting in a training cohort of 300 patients and a validation cohort of 128 patients with HER2-positive breast cancer, as well as a training cohort of 170 patients and a validation cohort of 73 patients with TNBC. Comparative analysis of clinicopathological characteristics between the training and validation cohorts demonstrated no statistically significant differences across all variables (*p* > 0.05 for all comparisons), confirming the comparability of the two datasets (Table 1, Supplementary Table 1).

By collecting pre- and post-NAT ultrasound imaging data from HER2-positive breast cancer and TNBC patients, we systematically analyzed the changes in the following parameters: maximum tumor diameter, tumor remission rate, peritumoral echogenicity, posterior echogenicity, tumor CDFI signal, lymph node remission rate, lymph node CDFI signal and hyperechoic medulla visible.

Among HER2-positive breast cancer patients, 292 cases (68.2%) achieved PR, 19 cases (4.4%) achieved CR, and only 4 cases (0.9%) exhibited PD following NAT. In TNBC, 141 cases (58.0%) showed PR, 11 cases (4.5%) achieved CR, and only 3 cases (1.2%) developed PD post-NAT. A comparison of ultrasonographic characteristics between the training and validation sets revealed no statistically significant differences in any parameters (all *p*>0.05), confirming the comparability of the two cohorts (Table 2, Supplementary Table 2).

### Clinicopathological and ultrasonographic factors influencing ypN0 status in HER2-Positive and TNBC

In the HER2-positive breast cancer training cohort (Table 3), 169 patients (56.3%) achieved ypN0, whereas 131 patients (43.7%) exhibited persistent ypN+. In the TNBC training cohort (Supplementary Table 3), 79 patients (46.5%) were classified as ypN0, and 91 patients (53.5%) remained ypN+.

In HER2-positive breast cancer, clinical N stage (*p*=0.009), histologic grade (*p*<0.001), ER expression status (*p*=0.001), PR expression status (*p*<0.001), HER2 expression status (*p*=0.001), presence of lymphovascular invasion (*p* = 0.002), NAT regimens incorporating dual-targeted therapy (*p*=0.029) or anthracycline-based combinations (*p*=0.036), and postoperative pCR in the primary breast tumor (*p* < 0.001) were significantly associated with achieving ypN0 following NAT (Table 3).

In TNBC, clinical N stage (*p*<0.001), histologic grade (*p*=0.003), p53 expression status (*p*=0.041), presence of lymphovascular invasion (*p*=0.022), NAT regimen (*p* = 0.002), NAT cycle duration (*p*=0.008), and postoperative pCR in the primary breast tumor (*p*<0.001) emerged as independent predictors of ypN0 post-NAT (Supplementary Table 3).

Univariate analysis was performed to compare imaging characteristics between the ypN0 and ypN+ groups. In HER2-positive breast cancer, the following factors were significantly associated with achieving ypN0 after NAT: post-NAT maximum tumor diameter, tumor remission rate post-NAT, peritumoral echogenicity post-NAT and changes in posterior echogenicity pre- and post-NAT, tumor CDFI signal patterns pre- to post-NAT, minimal transverse lymph node diameter post-NAT, lymph node remission rate post-NAT, visibility of hyperechoic medulla in axillary lymph nodes post-NAT and therapeutic response evaluation of the primary breast tumor (Table 4).

In TNBC, the critical predictors of ypN0 included: post-NAT maximum tumor diameter, tumor remission rate post-NAT, peritumoral echogenicity post-NAT and dynamic changes in peritumoral echogenicity pre- to post-NAT, post-NAT tumor CDFI signal and tumor CDFI signal alterations pre- and post-NAT, minimal transverse lymph node diameter pre- and post-NAT and lymph node remission rate post-NAT, post-NAT lymph node CDFI signal and lymph node CDFI signal evolution pre- to post-NAT and therapeutic response of the primary breast tumor (Supplementary Table 4).

### Multivariable analysis of factors associated with achieving ypN0 status in axillary lymph nodes following NAT in HER2-Positive and TNBC

The aforementioned significant clinicpathological and ultrasonographic factors affecting ypN0 status after NAT were incorporated into multivariate logistic regression analysis to further identify more robust independent predictive factors. The results demonstrated that HER2-positive breast cancer patients were more likely to achieve ypN0 status after NAT when achieving breast pCR, presenting with histologic grade III tumors, exhibiting HER2 IHC 3+ expression, lacking lymphovascular invasion, demonstrating clinical N1 stage, and showing prominent and hypervascular tumor CDFI signal pre-NAT (Table 5). Conversely, TNBC patients showed higher probability of achieving ypN0 status when attaining breast pCR, presenting histologic grade III tumors, receiving taxane combined with platinum-based regimens, displaying dot-linear lymph node CDFI signal post-NAT and having minimal transverse diameter of lymph node post-NAT (Table 6).

### Construction of the predictive model for the probability of achieving ypN0 status after NAT

**Figure 2:**
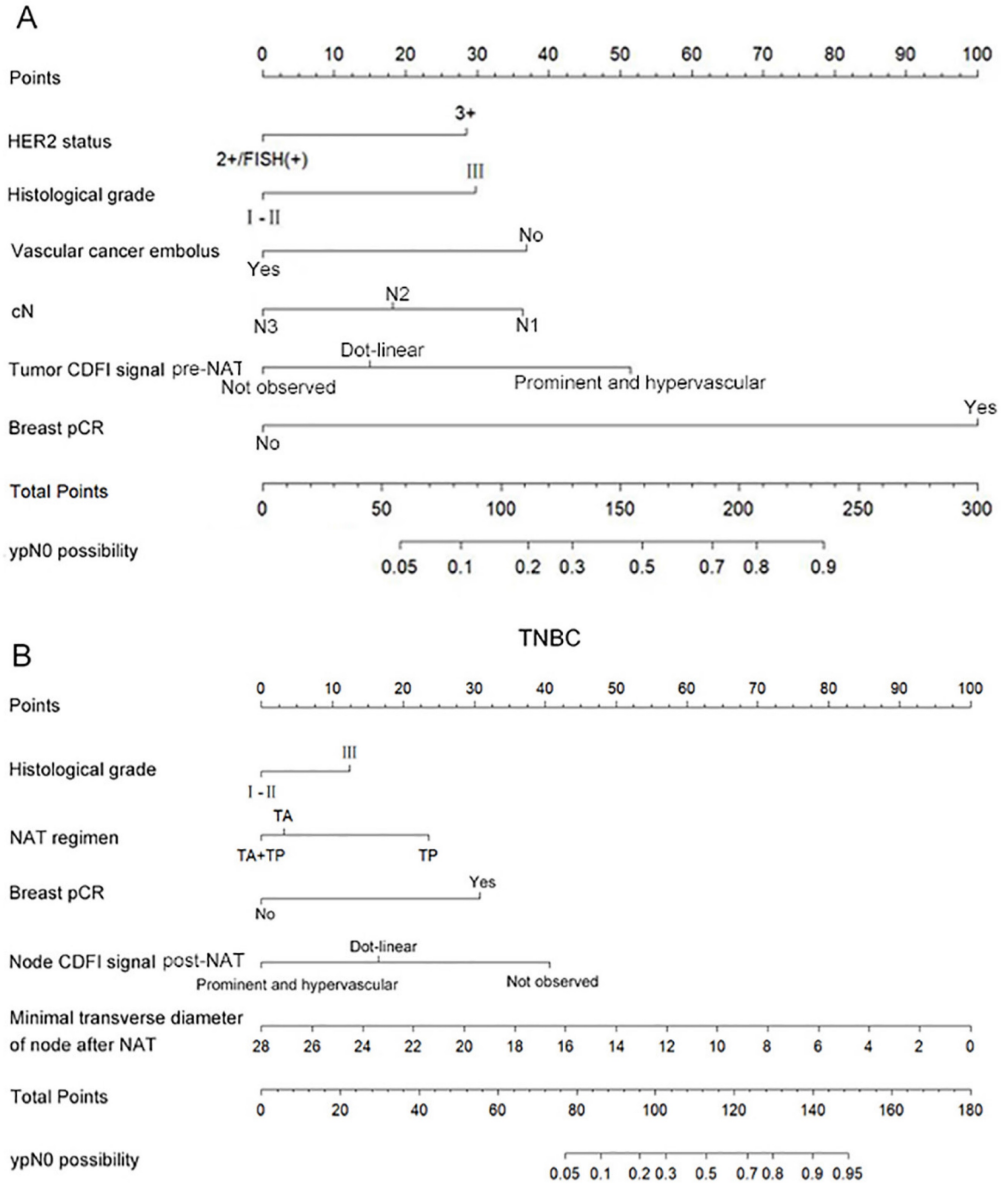
Nomogram to predict the probability of achieving ypN0 status after NAT for initially diagnosed pN+ patients with HER2 positive breast cancer (A) and TNBC (B).

Based on the multivariate analysis results identifying six significant independent predictors in HER2-positive breast cancer and five in TNBC, we developed predictive models to estimate the probability of achieving ypN0 status after NAT for initially diagnosed pN+ HER2-positive and TNBC patients. These models were visualized through nomograms constructed with R software (Figure 2A, B). The nomogram projects each predictor onto a standardized scale, quantifying individual contributions as discrete points. By summing these points across all variables, a total score is generated. This score is then mapped to a probability axis, enabling clinicians to directly estimate the likelihood of ypN0 attainment based on cumulative predictor profiles.

### Evaluation and validation of predictive model performance

**Figure 3:**
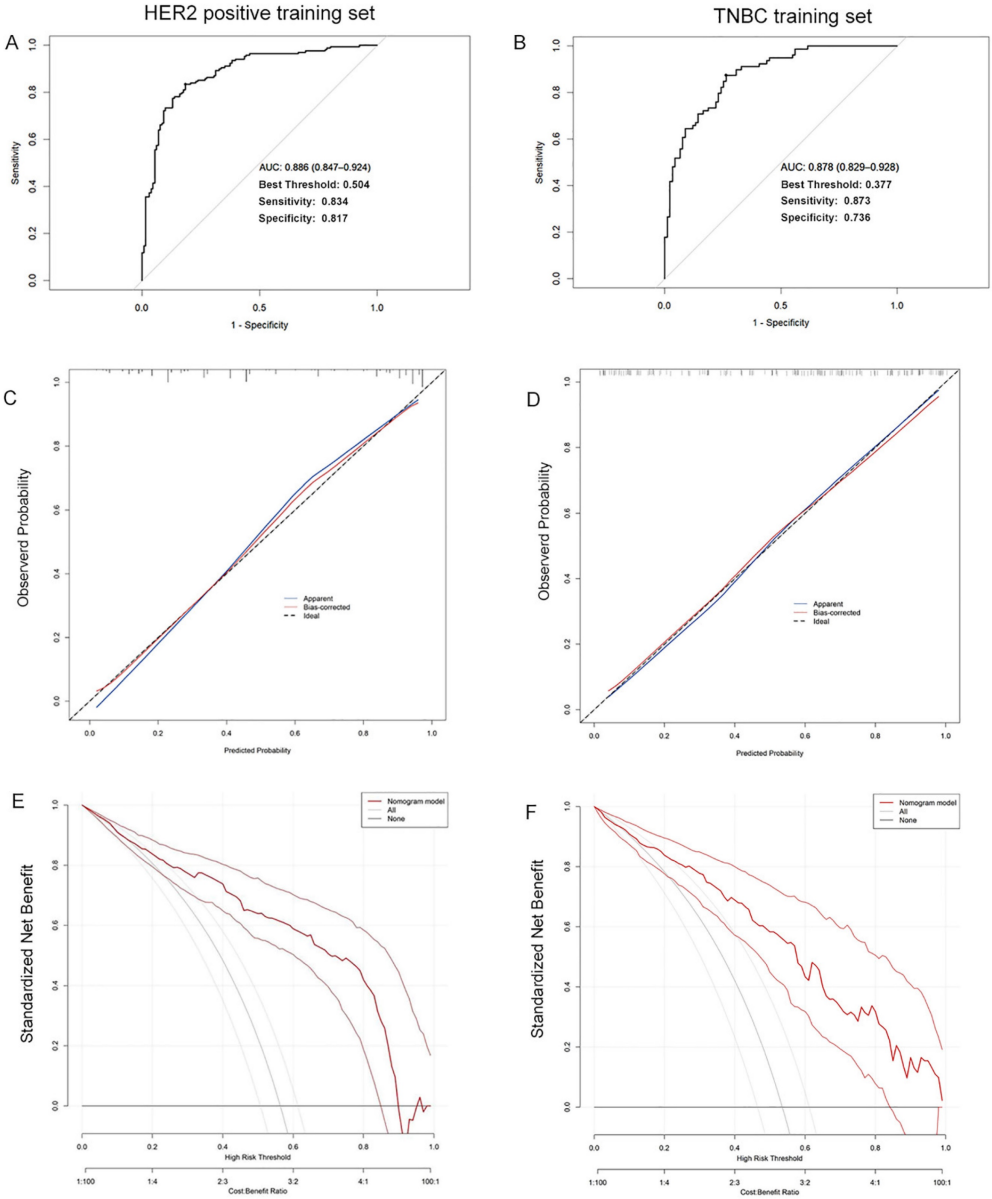
The ROC curves (A), calibration curves (B), and decision curves (C) are shown for the prediction model in the training cohort of HER2 positive breast cancer and TNBC.

According to the predictive model, ROC curves were plotted for the training sets of HER2-positive breast cancer and TNBC, with calculated AUC values of 0.886 (95% CI: 0.847-0.924) and 0.878 (95% CI: 0.829-0.928), respectively. The optimal cutoff values were 0.504 and 0.377, with sensitivities of 0.834 and 0.873, and specificities of 0.817 and 0.736, indicating favorable predictive performance and high discriminative ability of the models (Figure 3A, 3B). Calibration curves based on the predictive models demonstrated moderate agreement between the model-predicted outcomes and the calibration diagonal line, suggesting good consistency between the predicted and actual results and high calibration accuracy (Figure 3C, 3D). Finally, decision curves derived from the predictive models showed that the model's net benefit curve lay above both the "None" and "All" lines across most threshold ranges. This indicates that the model's net benefit rate surpassed the simplistic strategies of either diagnosing all patients as not achieving ypN0 or all achieving ypN0, highlighting its potential clinical utility (Figure 3E, 3F).

**Figure 4:**
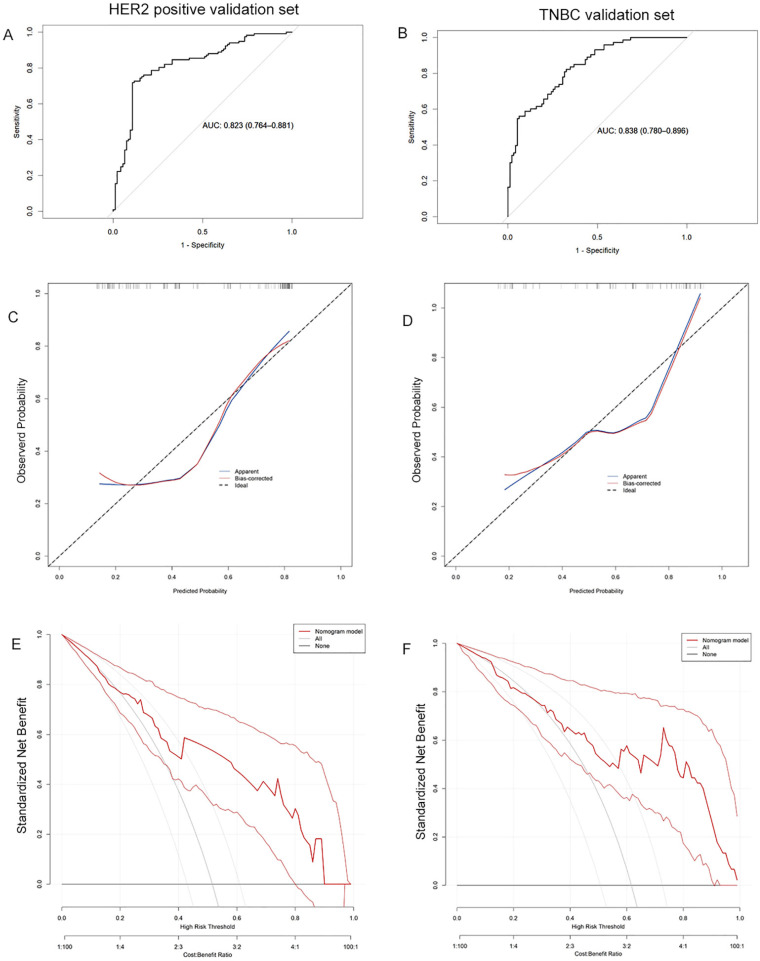
The ROC curves (A), calibration curves (B), and decision curves (C) are shown for the prediction model in the validation cohort of HER2 positive breast cancer and TNBC.

The validation cohort was subsequently applied to the model, with ROC curves, calibration curves, and decision curves analysis generated accordingly. The calculated AUC values were 0.823 (95% CI: 0.764-0.881) for HER2-positive breast cancer and 0.838 (95% CI: 0.780-0.896) for TNBC (Figure 4A, 4B). Calibration curves remained closely aligned with the ideal diagonal across both subtypes (Figure 4C, 4D). Furthermore, the decision curves demonstrated superior net benefit rates over the "None" and "All" strategies across a wide threshold range (Figure 4E, 4F). These results confirm the model's sustained predictive accuracy, robust calibration, and enhanced clinical utility in the validation cohort, underscoring its reliability for practical application.

## Discussion

Axillary lymph node status remains one of the most critical prognostic factors in breast cancer, with pN+ patients typically demonstrating higher recurrence rates and shorter survival durations. With continuous improvements in NAT efficacy, a substantial proportion of initially pN+ patients achieve ypN0 status post-NAT. Consequently, growing research attention has focused on this patient cohort exhibiting pN+ to ypN0 conversion, aiming to expand eligibility criteria for omitting ALND, mitigate overtreatment, and ultimately reduce surgical morbidity while enhancing postoperative quality of life.

Multiple large-scale meta-analyses have conclusively demonstrated that HER2-positive and TNBC breast cancer exhibit significantly higher sensitivity to NAT compared to other molecular subtypes, a feature intrinsically linked to patient prognosis 19, 20. Notably, the conversion from initially diagnosed cN+ to ypN0 status post-NAT occurs more frequently in these two subtypes. These findings informed our stratified investigation focusing specifically on HER2-positive and TNBC cohorts. In our study, 54.9% of HER2-positive and 44.0% of TNBC patients achieved ypN0 status following NAT, rates substantially exceeding their respective breast pCR rates (43.4% for HER2-positive vs. 27.5% for TNBC). This discrepancy not only underscores the enhanced systemic responsiveness of these subtypes to NAT but also highlights the dissociation between primary tumor response and nodal clearance. The higher prevalence of ypN0 achievement compared to breast pCR further suggests a clinically actionable opportunity for ALND omission in a substantial proportion of these patients.

Extensive evidence demonstrates a strong association between breast pCR and axillary nodal status following NAT in HER2-positive and TNBC, whereas this correlation remains attenuated in HR-positive/HER2-negative subtypes 21. A landmark Mayo Clinic study analyzing over 30,000 cases established that HER2-positive and TNBC patients with initial cN0-1 status exhibit the highest likelihood of achieving breast pCR post-NAT, with ypN+ rates below 2% among those attaining pCR 22. Our findings further corroborate this relationship: in the HER2-positive cohort, 87.1% (162/186) of patients achieving breast pCR concurrently attained ypN0 status, while 77.6% (52/67) of TNBC patients with breast pCR demonstrated nodal clearance. Multivariate analysis confirmed breast pCR as an independent predictor of ypN0 achievement in both subtypes (HER2-positive: OR 6.24, 95% CI 3.02-12.91; TNBC: OR 4.78, 95% CI 2.15-10.62). These results strongly suggest that HER2-positive and TNBC patients attaining breast pCR after NAT harbor exceptionally high probabilities of axillary pathologic downstaging (pN+→ypN0), thereby supporting the clinical rationale for omitting ALND in this responsive population.

HER2 serves as a pivotal therapeutic target in breast cancer, with evolving anti-HER2 agents substantially improving prognoses for HER2-positive patients. This study identified HER2 expression status as an independent predictor of ypN0 achievement following NAT in the HER2-positive subgroup, where patients with IHC 3+ tumors exhibited significantly higher ypN0 rates compared to those with IHC 2+/FISH+ status. These findings align with large-scale investigations such as the CSBrS-026 trial 23, 24, suggesting enhanced sensitivity to anti-HER2 therapies in tumors with HER2 protein overexpression (IHC 3+). In HR-positive/HER2-positive tumors, however, crosstalk between HR and HER2 signaling pathways may attenuate treatment efficacy. Notably, Atallah et al. demonstrated that HER2 IHC 3+ could override this antagonistic interplay, achieving comparable pCR rates between HR-positive and HR-negative subgroups 24. Within the TNBC cohort, patients receiving taxane-platinum combination regimens showed superior ypN0 attainment compared to those treated with anthracycline-taxane protocols. This underscores the therapeutic advantage of platinum-based chemotherapy in TNBC, corroborated by meta-analyses showing significantly elevated pCR rates with platinum inclusion 25, 26. The differential response may stem from platinum's preferential targeting of homologous recombination-deficient TNBC subsets. Recent evidence has demonstrated that the addition of neoadjuvant pembrolizumab to chemotherapy significantly increases pCR rates and prolongs event-free survival compared to NAT alone in TNBC patients. Consequently, pembrolizumab (anti-PD-1) combined with chemotherapy has now been established as the standard NAT regimen for TNBC 27.

Furthermore, dynamic tumor response during treatment harbors critical predictive information, with imaging modalities providing real-time assessment of therapeutic changes across NAT cycles. Emerging evidence highlights that integrating baseline clinicopathological characteristics with longitudinal imaging features enhances the characterization of tumor temporal heterogeneity and NAT responsiveness, thereby improving outcome prediction accuracy 28. Although MRI holds certain advantages in assessing neoadjuvant therapy response for breast cancer, breast MRI examinations are relatively costly. Additionally, patients with metallic implants or claustrophobia cannot undergo MRI. Consequently, not all enrolled patients in this study underwent breast MRI before and after neoadjuvant therapy. For these reasons, MRI indicators were not selected as imaging factors for the predictive model. Compared to mammography, MRI, and PET-CT, ultrasound offers distinct advantages as a non-irradiating, cost-effective, and highly reproducible modality accessible to most patients. Our findings reveal that pre-NAT tumor vascularity on color CDFI, post-NAT CDFI patterns, and their longitudinal changes significantly influence ypN0 attainment in HER2-positive breast cancer. In subsequent analyses, the CDFI signals of the tumor prior to NAT were identified as an independent influencing factor.

Patients with tumors exhibiting prominent and hypervascular blood flow signals on CDFI were more likely to achieve ypN0 compared with those whose tumors showed dot-linear blood flow signals, or no blood flow signals at all. This may be because tumors with abundant CDFI signals prior to NAT, indicating rich blood supply and active angiogenesis, are more likely to be rapidly proliferating and thus more sensitive to NAT 29. These observations align with prior studies validating CDFI's predictive value for pCR post-NAT 30, 31. In TNBC, minimal transverse diameter of lymph node and lymph node CDFI signal patterns post-NAT emerged as independent predictors. Notably, patients with a smaller minimum diameter of axillary lymph nodes and those with dot-linear CDFI signals in the axillary lymph nodes were most likely to achieve ypN0. A previous study indicated that axillary metastatic lymph nodes are prone to undergo degeneration following NAT, which may result in their replacement by normal tissue cells, and even manifest as collagenization or fibrosis. Consequently, the ultrasonographic features of these lymph nodes, such as shape, size, cortical thickness, and blood flow signals, may also undergo corresponding changes. These changes can be utilized to further evaluate the status of axillary lymph nodes after NAT 32. For instance, the ACOSOG Z1071 trial 33 demonstrated that incorporating such nodal ultrasound characteristics significantly reduced SLNB false-negative rates, underscoring their clinical utility in axillary restaging.

In summary, this study systematically integrated baseline clinicopathological characteristics with pre- and post- NAT ultrasonographic features of primary tumors and axillary lymph nodes in HER2-positive and TNBC cohorts. We identified predictive factors for axillary pathologic downstaging (pN+→ypN0) and developed subtype-specific predictive models, subsequently visualized and validated through nomograms. However, several limitations warrant consideration. First, the retrospective single-center design introduced potential selection bias, particularly in NAT regimen allocation influenced by regional drug availability and socioeconomic constraints. Specifically, pertuzumab for NAT was not included in China's National Reimbursement Drug List until the 2021 update. Prior to this, patients were required to fully self-fund pertuzumab. As a result, a subset of HER2-positive patients in this study received single trastuzumab-based therapy combined with chemotherapy due to the high cost of pertuzumab. Furthermore, due to the current lack of coverage for BRCA1/2 mutation testing under China's basic medical insurance scheme, the associated costs remain relatively high. Consequently, testing rates among the TNBC in this study were low. BRCA1/2 mutation status data were not systematically collected. Second, the operator-dependent nature of ultrasound interpretation and incomplete documentation of advanced parameters (e.g., tumor elasticity scores, nodal cortical thickness, hilar architecture) limited longitudinal comparisons and feature incorporation. Future multicenter prospective studies incorporating standardized imaging protocols, molecular profiling, and socioeconomic variables are required to refine nomogram generalizability. Additionally, this study aimed to develop a nomogram for predicting axillary pCR after NAT. Therefore, the dataset used in this analysis did not include long-term follow-up data. Despite these constraints, our findings align with the precision medicine paradigm prioritizing treatment de-escalation without compromising oncologic safety. In this retrospective study, while data on bpCR can be extracted from postoperative pathology reports, clinical determination of bpCR achievement may be ascertained through image-guided vacuum-assisted core biopsy (VACB) 34, 35. The proposed models provide clinicians with a practical tool to stratify patients likely to achieve ypN0 status post-NAT, thereby informing shared decision-making regarding axillary surgery omission. Furthermore, these results establish a preliminary evidence base for integrating dynamic ultrasonographic biomarkers into therapeutic response assessment frameworks, potentially guiding personalized NAT optimization in biologically aggressive subtypes.

## Supplementary Material

Supplementary tables.

## Figures and Tables

**Table 1:** Clinicopathological characteristics of the training set and the validation set in HER2-positive breast cancer

Clinicopathological characteristics	Total (n=428)	Training set n (%)	Validation set n (%)	χ²	*p* value
**Age(years)**				1.133	0.292
≤50	194	141 (47.0)	53 (41.4)		
>50	234	159 (53.0)	75 (58.6)		
**Location of tumor**				0.023	0.916
Left breast	235	164 (54.7)	71 (55.5)		
Right breast	193	136 (45.3)	57 (44.5)		
**Surgery**				0.343	0.607
Mastectomy	411	287 (95.7)	124 (96.9)		
Breast-conserving	17	13 (4.3)	4 (3.1)		
**Menstrual status**				1.379	0.245
Premenopausal	189	138 (46.0)	51 (39.8)		
Postmenopausal	239	162 (54.0)	77 (60.2)		
**Pregnancies number**				2.139	0.343
0	13	11 (3.7)	2 (1.6)		
1, 2, 3	343	242 (80.7)	101 (78.9)		
≥4	72	47 (15.6)	25 (19.5)		
**cT**				4.650	0.199
T1	31	23 (7.7)	8 (6.2)		
T2	245	173 (57.7)	72 (56.3)		
T3	135	96 (32.0)	39 (30.5)		
T4	17	8 (2.6)	8 (7.0)		
**cN**				0.326	0.849
N1	217	151 (50.3)	66 (51.6)		
N2	108	78 (26.0)	30 (23.4)		
N3	103	71 (23.7)	32 (25.0)		
**Histological type**				0.051	1.000
IDC	419	294 (98.0)	125 (97.7)		
Others	9	6 (2.0)	3 (2.3)		
**Histological grade**				0.307	0.598
I-II	232	160 (53.3)	72 (56.3)		
III	196	140 (46.7)	56 (43.7)		
**TILs**				0.000	1.000
<10%	321	225 (75.0)	96 (75.0)		
≥10%	107	75 (25.0)	32 (25.0)		
**ER status**				0.015	0.916
Negative	192	134 (44.7)	58 (45.3)		
Positive	236	166 (55.3)	70 (54.7)		
**PR status**				0.916	0.361
Negative	297	204 (68.0)	93 (72.7)		
Positive	131	96 (32.0)	35 (27.3)		
**HER2 status**				0.313	0.609
IHC2+/FISH+	93	63 (21.0)	30 (23.4)		
IHC 3+	335	237 (79.0)	98 (76.6)		
**Ki-67 expression**				3.440	0.112
<20%	8	6 (2.0)	2 (1.6)		
≥20%	420	294 (98.0)	126 (98.4)		
**p53 expression**				0.012	0.916
Negative	199	140 (46.7)	59 (46.1)		
Positive	229	160 (53.3)	69 (53.9)		
**CK56 expression**				0.624	0.452
Negative	383	275 (91.7)	108 (84.4)		
Positive	45	25 (8.3)	20 (15.6)		
**EGFR exprssion**				0.014	0.914
Negative	266	187 (63.3)	79 (61.7)		
Positive	162	113 (37.7)	49 (38.3)		
**AR expression**				1.821	0.195
Negative	38	23 (7.7)	15 (11.7)		
Positive	390	277 (92.3)	113 (88.3)		
**Lymphovascular invasion**				0.299	0.641
No	372	259 (86.3)	113 (88.3)		
Yes	56	41 (13.7)	15 (11.7)		
**Breast pCR**				0.006	1.000
pCR	186	130 (43.3)	56 (43.7)		
non-pCR	242	170 (56.7)	72 (56.3)		
**Axillary lynph node status**				0.825	0.397
ypN0	235	169 (56.3)	66 (51.6)		
ypN+	193	131 (43.7)	62 (48.4)		
**Applicaton of targeted therapy**				0.012	1.000
Single-target therapy	59	41 (13.7)	18 (14.1)		
Dual-target therapy	369	259 (86.3)	110 (85.9)		
**Applicaion of anthracycline**				0.263	0.652
No	290	201 (67.0)	89 (69.5)		
Yes	138	99 (33.0)	39 (30.5)		
**NAT cycles**				0.573	0.477
4	69	51 (17.0)	18 (14.1)		
>4	359	249 (83.0)	110 (85.9)		

**Table 2:** Ultrasonographic characteristics of the training set and the validation set in HER2-positive breast cancer pre- and post-NAT

Ultrasonographic characteristics	Total (n=428)	Training set, n (%)	Validation set, n (%)	t/χ²/Z	*p* value
**Maximum tumor diameter (mm)**					
Before NAT		42 (10, 128)	42 (12,160)	-0.686	0.493
After NAT		21 (0, 80)	21 (0, 100)	-0.213	0.902
**Tumor remission rate (%, mean ± SD)**		44.03±24.68	45.99±26.16	-0.709	0.460
**RECIST**				2.974	0.396
CR	19	10 (3.3)	9 (7.0)		
PR	292	206 (68.7)	86 (67.2)		
SD	113	81 (27.0)	32 (25.0)		
PD	4	3 (1.0)	1 (0.8)		
**Peritumoral echogenicity pre-NAT**				0.208	0.702
Enhancement	394	275 (91.7)	119 (93.0)		
Unremarkable	34	25 (8.3)	9 (7.0)		
**Peritumoral echogenicity post-NAT**				0.546	0.477
Enhancement	314	217 (72.3)	97 (75.8)		
Unremarkable	114	83 (27.7)	31 (24.2)		
**Peritumoral echogenicity changes**				0.205	0.694
Unchanged	342	238 (79.3)	104 (81.3)		
Changed	86	62 (20.7)	24 (18.7)		
**Posterior echogenicity pre-NAT**				1.281	0.527
Unremarkable	173	125 (41.7)	48 (37.5)		
Attenuation	249	170 (56.7)	79 (61.7)		
Enhancement	6	5 (1.6)	1 (0.8)		
**Posterior echogenicity post-NAT**				3.407	0.182
Unremarkable	300	215 (71.7)	85 (66.4)		
Attenuation	124	81 (27.0)	43 (33.6)		
Enhancement	4	4 (1.3)	0		
**Posterior echogenicity changes**				0.051	0.908
Unchanged	301	210 (70.0)	91 (71.1)		
Changed	127	90 (30.0)	37 (28.9)		
**Tumor CDFI signal pre-NAT**				0.387	0.824
Prominent and hypervascular	344	240 (80)	104 (81.3)		
Dot-linear	45	31(10.3)	13 (10.9)		
Not observed	39	29 (9.7)	10 (7.8)		
**Tumor CDFI signal post-NAT**				2.368	0.306
Prominent and hypervascular	180	121 (40.3)	59 (46.1)		
Dot-linear	60	40 (13.3)	20 (15.6)		
Not observed	188	139 (46.4)	49 (38.3)		
**Tumor CDFI signal changes**				0.829	0.661
Unchanged	220	152 (50.7)	68 (53.1)		
Reduced	195	140 (46.7)	55 (43.0)		
Increased	13	8 (2.6)	5 (3.9)		
**Minimal transverse diameter of lymph node (mm)**					
Before NAT		11 (3, 52)	11 (5, 44)	-0.492	0.623
After NAT		6 (0, 19)	6 (0, 20)	-0.133	0.894
**Lymph node remission rate (%)**		50 (-66.67, 100)	50 (-27.27, 100)	-0.517	0.605
**Lymph node CDFI signal pre-NAT**				1.499	0.473
Prominent and hypervascular	202	136 (45.3)	66 (51.6)		
Dot-linear	47	35 (11.7)	12 (9.3)		
Not observed	179	129 (43.0)	50 (39.1)		
**Lymph node CDFI signal post-NAT**				2.490	0.288
Prominent and hypervascular	94	66 (22.0)	28 (21.9)		
Dot-linear	49	39 (13.0)	10 (7.8)		
Not observed	285	195 (65.0)	90 (70.3)		
**Lymph node CDFI signal changes**				4.545	0.103
Unchanged	290	209 (69.7)	81 (63.3)		
Reduced	133	86 (28.7)	47 (36.7)		
Increased	5	5 (1.6)	0		
**Hyperechoic medulla visible pre-NAT**				0.246	0.638
No	423	297 (99.0)	126 (98.4)		
Yes	5	3 (1.0)	2 (1.6)		
**Hyperechoic medulla visible post-NAT**				1.222	0.328
No	394	279 (93.0)	115 (89.8)		
Yes	34	21 (7.0)	113 (10.2)		

**Table 3:** Univariate analysis of clinicopathological characteristics influencing the achievement of ypN0 status in HER2-positive breast cancer in training set

Clinicopathological characteristics	Total (n=300)	ypN0, n (%)	ypN+, n (%)	χ²	*p* value
**Age(years)**				0.111	0.816
≤50	141	78 (46.2)	63 (48.1)		
>50	159	91 (53.8)	68 (51.9)		
**Location of tumor**				0.021	0.907
Left breast	164	93 (55.0)	71 (54.2)		
Right breast	136	76 (45.0)	60 (45.8)		
**Surgery**				0.150	0.781
Mastectomy	287	161 (95.3)	126 (96.2)		
Breast-conserving	13	8 (4.7)	5 (3.8)		
**Menstrual status**				0.004	1.000
Premenopausal	138	78 (46.2)	60 (45.8)		
Postmenopausal	162	91 (53.8)	71 (54.2)		
**Pregnancies number**				1.316	0.518
0	11	8 (4.7)	3 (2.3)		
1, 2, 3	242	134 (79.3)	108 (82.4)		
≥4	47	27 (16.0)	20 (15.3)		
**cT**				5.404	0.145
T1	23	11 (6.5)	12 (9.2)		
T2	173	96 (56.8)	77 (58.8)		
T3	96	60 (35.5)	36 (27.5)		
T4	8	2 (1.2)	6 (4.5)		
**cN**				9.439	0.009
N1	217	151 (50.3)	66 (51.6)		
N2	108	78 (26.0)	30 (23.4)		
N3	103	71 (23.7)	32 (25.0)		
**Histological type**				0.266	0.699
**IDC**	294	165 (97.6)	129 (98.5)		
**Others**	6	4 (2.4)	2 (1.5)		
**Histological grade**				14.171	<0.001
I-II	160	74 (43.8)	86 (65.6)		
III	140	95 (56.2)	45 (34.4)		
**TILs**				0.547	0.503
<10%	225	124 (73.4)	101 (77.1)		
≥10%	75	45 (26.6)	30 (22.9)		
**ER status**				11.549	0.001
Negative	134	90 (53.3)	44 (33.6)		
Positive	166	79 (46.7)	87 (66.4)		
**PR status**				20.356	<0.001
Negative	204	133 (78.7)	71 (54.2)		
Positive	96	36 (21.3)	60 (45.8)		
**HER2 status**				12.742	0.001
IHC2+/FISH+	63	23 (13.6)	40 (30.5)		
IHC 3+	237	146 (86.4)	91 (69.5)		
**Ki-67 expression**				2.597	0.190
<20%	6	1 (0.6)	5 (3.8)		
≥20%	294	168 (99.4)	126 (96.2)		
**p53 expression**				0.814	0.414
Negative	140	75 (44.4)	65 (49.6)		
Positive	260	94 (55.6)	66 (50.4)		
**CK56 expression**				1.686	0.212
Negative	275	158 (93.5)	117 (89.3)		
Positive	25	11 (6.5)	14 (10.7)		
**EGFR exprssion**				1.089	0.337
Negative	187	101 (59.8)	86 (65.6)		
Positive	113	68 (40.2)	45 (34.4)		
**AR expression**				0.733	0.512
Negative	23	11 (6.5)	12 (9.2)		
Positive	277	158 (93.5)	119 (90.8)		
**Lymphovascular invasion**				9.504	0.002
No	259	155 (91.7)	104 (79.4)		
Yes	41	14 (8.3)	27 (20.6)		
**Breast pCR**				100.931	<0.001
pCR	130	116 (68.6)	14 (10.7)		
non-pCR	170	53 (31.4)	117 (89.3)		
**Applicaton of targeted therapy**				4.269	0.029
Single-target therapy	41	17 (10.1)	24 (18.3)		
Dual-target therapy	259	152 (89.9)	107 (81.7)		
**Applicaion of anthracycline**				3.7	0.036
No	201	121 (71.6)	80 (61.1)		
Yes	99	48 (28.4)	51 (38.9)		
**NAT cycles**				0.287	0.643
4	51	27 (16.0)	24 (18.3)		
>4	249	142 (84.0)	107 (81.7)		

**Table 4:** Univariate analysis of ultrasonographic characteristics influencing the achievement of ypN0 status in HER2-positive breast cancer in training set

Ultrasonographic characteristics	Total (n=300)	ypN0, n (%)	ypN+, n (%)	t/χ²/Z	*p* value
**Maximum tumor diameter (mm)**					
Before NAT		42 (12, 97)	42 (10,128)	-0.460	0.646
After NAT		19 (0, 64)	24 (0, 80)	-3.791	<0.001
**Tumor remission rate (%, mean ± SD)**		49.22±24.56	37.32±23.27	4.257	<0.001
**RECIST**				15.066	0.002
CR	10	8 (4.7)	2 (1.5)		
PR	206	128 (75.7)	78 (59.5)		
SD	81	32 (18.9)	49 (37.5)		
PD	3	1 (0.6)	2 (1.5)		
**Peritumoral echogenicity pre-NAT**				0.149	0.834
Enhancement	275	154 (91.1)	121 (92.4)		
Unremarkable	25	15 (8.9)	10 (7.6)		
**Peritumoral echogenicity post-NAT**				4.601	0.037
Enhancement	217	114 (67.5)	103 (78.6)		
Unremarkable	83	55 (32.5)	28 (21.4)		
**Peritumoral echogenicity changes**				6.804	0.010
Unchanged	238	125 (74.0)	113 (86.3)		
Changed	62	44 (26.0)	18 (13.7)		
**Posterior echogenicity pre-NAT**				3.247	0.197
Unremarkable	125	76 (45.0)	49 (37.4)		
Attenuation	170	89 (52.6)	81 (61.8)		
Enhancement	5	4 (2.4)	1 (0.8)		
**Posterior echogenicity post-NAT**				3.427	0.180
Unremarkable	215	127 (75.1)	88 (67.1)		
Attenuation	81	39 (23.1)	42 (32.1)		
Enhancement	4	3 (1.8)	1 (0.8)		
**Posterior echogenicity changes**				0.006	1.000
Unchanged	210	118 (69.8)	92 (70.2)		
Changed	90	51 (30.2)	39 (29.9)		
**Tumor CDFI signal pre-NAT**				8.232	0.016
Prominent and hypervascular	240	145 (85.8)	95 (72.6)		
Dot-linear	31	13 (7.7)	18 (13.7)		
Not observed	29	11 (6.5)	18 (13.7)		
**Tumor CDFI signal post-NAT**				8.804	0.012
Prominent and hypervascular	121	59 (34.9)	62 (47.3)		
Dot-linear	40	19 (11.2)	21 (16.1)		
Not observed	139	91 (53.9)	48 (36.6)		
**Tumor CDFI signal changes**				19.272	<0.001
Unchanged	152	70 (41.4)	82 (62.6)		
Reduced	140	97 (57.4)	43 (32.8)		
Increased	8	2 (1.2)	6 (4.6)		
**Minimal transverse diameter of lymph node (mm)**					
Pre-NAT		11 (3, 35)	11 (5, 52)	-0.228	0.820
Post-NAT		5 (0, 13)	6 (0, 19)	-2.598	0.009
**Lymph node remission rate (%)**		53.85 (-33.33, 100)	50 (-16.67, 100)	-2.216	0.027
**Lymph node CDFI signal pre-NAT**				2.423	0.298
Prominent and hypervascular	136	74 (43.8)	62 (47.3)		
Dot-linear	35	24 (14.2)	11 (8.4)		
Not observed	129	71 (42.0)	58 (44.3)		
**Lymph node CDFI signal post-NAT**				2.289	0.318
Prominent and hypervascular	66	32 (18.9)	34 (26.0)		
Dot-linear	39	24 (14.2)	15 (11.4)		
Not observed	195	113 (66.9)	82 (62.6)		
**Lymph node CDFI signal changes**				2.436	0.296
Unchanged	209	113 (66.9)	96 (73.3)		
Reduced	86	54 (31.9)	32 (24.4)		
Increased	5	2 (1.2)	3 (2.3)		
**Hyperechoic medulla visible pre-NAT**				2.349	0.260
No	297	166 (98.2)	131 (100)		
Yes	3	3 (1.8)	0		
**Hyperechoic medulla visible post-NAT**				3.620	0.044
No	279	153 (90.5)	126 (96.2)		
Yes	21	16 (9.5)	5 (3.8)		

**Table 5:** Multivariate analysis of clinicopathological and ultrasonographic characteristics influencing the achievement of ypN0 status in HER2-positive breast cancer patients in training set

Clinicopathological and ultrasonographic characteristics	β	OR (95% CI)	*p* value
**Breast pCR**	2.889	17.979 (8.815-36.669)	<0.001
**Histological grade**	-0.866	0.421 (0.227-0.781)	0.006
**HER2 status**	-0.807	0.446 (0.213-0.937)	0.033
**lymphovascular invasion**	1.095	2.989 (1.230-7.260)	0.016
**cN**			0.017
N1	1	1	
N2	0.750	2.118 (1.021-4.393)	0.044
N3	1.005	2.733 (1.286-5.809)	0.009
**Tumor CDFI signal pre-NAT**			0.007
Prominent and hypervascular	1	1	
Dot-linear	1.047	2.850 (1.025-7.927)	0.045
Not observed	1.513	4.450 (1.516-13.589)	0.007

**Table 6:** Multivariate analysis of clinicopathological and ultrasonographic characteristics influencing the achievement of ypN0 status in TNBC patients in training set

Clinicopathological and ultrasonographic characteristics	β	OR (95% CI)	*p* value
**Breast pCR**	2.523	12.467 (4.308-36.081)	<0.001
**Histological grade**	-1.013	0.363 (0.155-0.850)	0.020
**NAT regimen**			0.037
TA	1	1	
TA→TP	0.254	1.289 (0.319-5.205)	0.721
TP	-1.675	0.187 (0.049-0.712)	0.014
**Minimal transverse diameter of lymph node post-NAT**	0.292	1.340 (1.145-1.568)	<0.001
**Lymph node CDFI signal post-NAT**			
Prominent and hypervascular	1	1	
Dot-linear	-3.331	0.036 (0.007-0.184)	<0.001
Not observed	-1.347	0.260 (0.081-0.831)	0.023

## Abbreviations

NAT: Neoadjuvant therapy
pN+: Pathologically confirmed axillary lymph node metastasis
ypN0: No residual nodal metastasis
ROC: Receiver operating characteristic
TNBC: Triple negative breast cancer
pCR: Pathological complete response
bpCR: Breast pathological complete response
ALND: Axillary lymph node dissection
SLNB: Sentinel lymph node biopsy
cN0: Clinically node-negative
RECIST: Response Evaluation Criteria in Solid Tumors
CR: Complete Response
PR: Partial Response
PD: Progressive Disease
SD: Stable Disease
CDFI: Color Doppler Flow Imaging
AUC: Area under the curve
VACB: vacuum-assisted core biopsy
