# Supplementary material for: Nomogram for predicting axillary pathologic complete response after neoadjuvant systemic therapy in HER2 positive and triple negative breast cancer
Source: J Cancer. 2025 Sep 22;16(13):3991–4007. doi: 10.7150/jca.118908 (PMC12491205; doi:10.7150/jca.118908)
Supplement: Supplementary file 1 — Supplementary tables. [file jcav16p3991s1.pdf]

Supplementary Table 1 Clinicopathological characteristics of the training set and the validation set in TNBC

| Clinicopathological characteristics | Total (n=243) | Training set n (%) | Validation set n (%) | $\chi^2$ | *p* value |
|---|---|---|---|---|---|
| **Age(years)** | | | | 0.057 | 0.889 |
| ≤50 | 117 | 81 (47.6) | 36 (49.3) | | |
| >50 | 126 | 89 (52.4) | 37 (50.7) | | |
| **Location of tumor** | | | | 2.259 | 0.133 |
| Left breast | 131 | 97 (57.1) | 34 (46.6) | | |
| Right breast | 112 | 73 (42.9) | 39 (53.4) | | |
| **Surgery** | | | | 2.346 | 0.138 |
| Mastectomy | 223 | 153 (90.0) | 70 (95.9) | | |
| Breast-conserving | 20 | 17 (10.0) | 3 (4.1) | | |
| **Menstrual status** | | | | 0.016 | 1.000 |
| Premenopausal | 118 | 83 (48.8) | 35 (47.9) | | |
| Postmenopausal | 125 | 87 (51.2) | 38 (52.1) | | |
| **Pregnancies number** | | | | 0.273 | 0.872 |
| 0 | 5 | 4 (2.4) | 1 (1.4) | | |
| 1, 2, 3 | 190 | 133 (78.2) | 57 (78.1) | | |
| ≥4 | 48 | 33 (19.4) | 15 (20.5) | | |
| **cT** | | | | 6.661 | 0.084 |
| T1 | 27 | 23 (13.5) | 4 (5.5) | | |
| T2 | 155 | 111 (65.4) | 44 (60.2) | | |
| T3 | 51 | 30 (17.6) | 21 (28.8) | | |
| T4 | 10 | 6 (3.5) | 4 (5.5) | | |
| **cN** | | | | 5.418 | 0.067 |
| N1 | 97 | 76 (44.7) | 21 (28.8) | | |
| N2 | 91 | 52 (30.6) | 39 (53.4) | | |
| N3 | 55 | 42 (24.7) | 13 (17.8) | | |
| **Histological type** | | | | 0.048 | 1.000 |
| IDC | 234 | 164 (96.5) | 70 (95.9) | | |
| Others | 9 | 6 (3.5) | 3 (4.1) | | |
| **Histological grade** | | | | 0.045 | 0.889 |
| I-II | 114 | 79 (46.5) | 35 (47.9) | | |
| III | 129 | 91 (53.5) | 38 (52.1) | | |
| **TILs** | | | | 0.051 | 0.883 |
| <10% | 159 | 112 (65.9) | 47 (64.4) | | |
| ≥10% | 84 | 58 (34.1) | 26 (35.6) | | |
| **HER2 status** | | | | 0.046 | 0.888 |
| IHC 0 | 104 | 72 (42.4) | 32 (43.8) | | |
| IHC 1+/ 2+FISH- | 139 | 98 (57.6) | 41 (56.2) | | |
| **Ki-67 expression** | | | | 0.923 | 0.458 |
| <20% | 9 | 5 (2.9) | 4 (5.5) | | |
| ≥20% | 234 | 165 (97.1) | 69 (94.5) | | |
| **p53 expression** | | | | 0.533 | 0.479 |
| Negative | 98 | 66 (38.8) | 32 (43.8) | | |
| Positive | 145 | 104 (61.2) | 41 (56.2) | | |
| **CK5/6 expression** | | | | 0.464 | 0.564 |
| Negative | 92 | 62 (36.5) | 30 (41.1) | | |
| Positive | 151 | 108 (63.5) | 43 (58.9) | | |
| **EGFR exprssion** | | | | 0.017 | 1.000 |
| Negative | 31 | 22 (12.9) | 9 (12.3) | | |
| Positive | 212 | 148 (87.1) | 64 (87.7) | | |
| **AR expression** | | | | 3.511 | 0.083 |
| Negative | 151 | 113 (66.5) | 38 (52.1) | | |
| Positive | 92 | 57 (33.5) | 35 (47.9) | | |
| **Lymphovascular invasion** | | | | 0.023 | 1.000 |
| No | 224 | 157 (92.4) | 67 (91.8) | | |
| Yes | 19 | 13 (7.6) | 6 (8.2) | | |
| **Breast pCR** | | | | 0.125 | 0.757 |
| pCR | 67 | 48 (28.2) | 19 (26.0) | | |
| non-pCR | 176 | 122 (71.8) | 54 (74.0) | | |
| **Axillary lynph node status** | | | | 1.364 | 0.262 |
| ypN0 | 107 | 79 (46.5) | 28 (38.4) | | |
| ypN+ | 136 | 91 (53.5) | 45 (61.6) | | |
| **NAT regimen** | | | | 0.259 | 0.897 |
| TA | 179 | 124 (72.9) | 55 (75.3) | | |
| TA→TP | 37 | 26 (15.3) | 11 (15.1) | | |
| TP | 27 | 20 (11.8) | 7 (9.6) | | |
| **NAT cycles** | | | | 0.285 | 0.719 |
| 4 | 45 | 30 (17.6) | 15 (20.5) | | |
| >4 | 198 | 140 (82.4) | 58 (79.5) | | |

Supplementary Table 2 Ultrasonographic characteristics of the training set and the validation set in TNBC pre- and post- NAT

| Ultrasonographic characteristics | Total (n=243) | Training set n (%) | Validation set n (%) | t/$\chi^2$/Z | *p* value |
|---|---|---|---|---|---|
| **Maximum tumor diameter (mm)** | | | | | |
| Before NAT | | 34 (11, 131) | 40 (10, 121) | 1.682 | 0.089 |
| After NAT | | 20 (0, 92) | 22 (0, 100) | -1.585 | 0.113 |
| **Tumor remission rate(%, mean±SD)** | | 38.46±28.87 | 36.33±24.27 | 0.553 | 0.581 |
| **RECIST** | | | | | |
| CR | 11 | 10 (5.9) | 1 (1.4) | 3.955 | 0.266 |
| PR | 141 | 98 (57.6) | 43 (58.9) | | |
| SD | 88 | 59 (34.7) | 29 (39.7) | | |
| PD | 3 | 3 (1.8) | 0 | | |
| **Peritumoral echogenicity pre-NAT** | | | | 0.873 | 1.000 |
| Enhancement | 211 | 148 (87.1) | 63 (86.3) | | |
| Unremarkable | 32 | 22 (12.9) | 10 (13.7) | | |
| **Peritumoral echogenicity post-NAT** | | | | 1.051 | 0.343 |
| Enhancement | 179 | 122 (71.8) | 57 (78.1) | | |
| Unremarkable | 64 | 48 (28.2) | 16 (21.9) | | |
| **Peritumoral echogenicity changes** | | | | 1.229 | 0.327 |
| Unchanged | 207 | 142 (83.5) | 65 (89.0) | | |
| Changed | 36 | 28 (16.5) | 8 (11.0) | | |
| **Posterior echogenicity pre-NAT** | | | | 1.763 | 0.414 |
| Unremarkable | 86 | 56 (32.9) | 30 (41.1) | | |
| Attenuation | 147 | 106 (62.4) | 41 (56.2) | | |
| Enhancement | 10 | 8 (4.7) | 2 (2.7) | | |
| **Posterior echogenicity post-NAT** | | | | 1.552 | 0.460 |
| Unremarkable | 159 | 107 (62.9) | 52 (71.2) | | |
| Attenuation | 8 | 60 (35.3) | 20 (27.4) | | |
| Enhancement | 4 | 3 (1.8) | 1 (1.4) | | |
| **Posterior echogenicity changes** | | | | 0.005 | 1.000 |
| Unchanged | 169 | 118 (69.4) | 51 (69.9) | | |
| Changed | 74 | 52 (30.6) | 22 (30.1) | | |
| **Tumor CDFI signal pre-NAT** | | | | 0.124 | 0.940 |
| **Prominent and hypervascular** | 183 | 127 (74.7) | 56 (76.7) | | |
| Dot-linear | 36 | 26 (15.3) | 10 (13.7) | | |
| Not observed | 24 | 17 (10.0) | 7 (9.6) | | |
| **Tumor CDFI signal post-NAT** | | | | 2.028 | 0.363 |
| Prominent and hypervascular | 101 | 66 (38.8) | 35 (47.9) | | |
| Dot-linear | 40 | 28 (16.5) | 12 (16.5) | | |
| Not observed | 102 | 76 (44.7) | 26 (35.6) | | |
| **Tumor CDFI signal changes** | | | | 2.442 | 0.295 |
| Unchanged | 128 | 84 (49.4) | 44 (60.3) | | |
| Reduced | 104 | 78 (45.9) | 26 (35.6) | | |
| Increased | 11 | 8 (4.7) | 3 (4.1) | | |
| **Minimal transverse diameter of lymph node (mm)** | | | | | |
| Pre-NAT | | 12 (3, 40) | 13 (4, 34) | -1.185 | 0.236 |
| Post-NAT | | 6 (0, 27) | 7 (0,25) | -0.819 | 0.413 |
| **Lymph node remission rate (%，mean±SD)** | | 42.89±28.35 | 44.62±27.01 | -0.443 | 0.658 |
| **Lymph node CDFI signal before NAT** | | | | 1.195 | 0.550 |
| Prominent and hypervascular | 121 | 82 (48.2) | 39 (53.4) | | |
| Dot-linear | 23 | 15 (8.9) | 8 (11.0) | | |
| Not observed | 99 | 73 (42.9) | 26 (35.6) | | |
| **Lymph node CDFI signal post-NAT** | | | | 0.316 | 0.854 |
| Prominent and hypervascular | 70 | 48 (28.2) | 22 (30.1) | | |
| Dot-linear | 27 | 18 (10.6) | 9 (12.3) | | |
| Not observed | 146 | 104 (61.2) | 42 (57.6) | | |
| **Lymph node CDFI signal changes** | | | | 0.367 | 0.832 |
| Unchanged | 168 | 118 (69.4) | 50 (68.5) | | |
| Reduced | 66 | 45 (26.5) | 21 (28.8) | | |
| Increased | 9 | 7 (4.1) | 2 (2.7) | | |
| **Hyperechoic medulla visible post-NAT** | | | | 0.500 | 0.524 |
| No | 233 | 162 (95.3) | 71 (97.3) | | |
| Yes | 10 | 8 (4.7) | 2 (2.7) | | |

Supplementary Table 3 Univariate analysis of clinicopathological characteristics influencing the achievement of ypN0 status in TNBC in training set

| Clinicopathological characteristics | Total (n=170) | ypN0 n (%) | ypN+ n (%) | $\chi^2$ | *p* value |
|---|---|---|---|---|---|
| **Age(years)** | | | | 0.175 | 0.758 |
| ≤50 | 81 | 39 (49.4) | 42 (46.2) | | |
| >50 | 89 | 40 (50.6) | 49 (53.8) | | |
| **Location of tumor** | | | | 0.825 | 0.438 |
| Left breast | 97 | 48 (60.8) | 49 (53.8) | | |
| Right breast | 73 | 31 (39.2) | 42 (46.2) | | |
| **Surgery** | | | | 0.003 | 1.000 |
| Mastectomy | 153 | 71 (89.9) | 82 (90.1) | | |
| Breast-conserving | 17 | 8 (10.1) | 9 (9.9) | | |
| **Menstrual status** | | | | 0.017 | 1.000 |
| Premenopausal | 83 | 39 (49.4) | 44 (48.4) | | |
| Postmenopausal | 87 | 40 (50.6) | 47 (51.6) | | |
| **Pregnancies number** | | | | 0.456 | 0.796 |
| 0 | 4 | 2 (2.5) | 2 (2.2) | | |
| 1, 2, 3 | 133 | 60 (75.9) | 73 (80.2) | | |
| ≥4 | 33 | 17 (21.5) | 16 (17.6) | | |
| **cT** | | | | 5.291 | 0.152 |
| T1 | 23 | 14 (17.7) | 9 (9.9) | | |
| T2 | 111 | 53 (67.1) | 58 (63.7) | | |
| T3 | 30 | 11 (13.9) | 19 (20.9) | | |
| T4 | 6 | 1 (1.3) | 5 (5.5) | | |
| **cN** | | | | 15.390 | < 0.001 |
| N1 | 76 | 48 (60.8) | 28 (30.8) | | |
| N2 | 52 | 17 (21.5) | 35 (38.4) | | |
| N3 | 42 | 14 (17.7) | 28 (30.8) | | |
| **Histological type** | | | | 2.221 | 0.217 |
| IDC | 164 | 78 (98.7) | 86 (94.5) | | |
| Others | 6 | 1 (1.3) | 5 (5.5) | | |
| **Histological grade** | | | | 8.966 | 0.003 |
| I-II | 79 | 27 (34.2) | 52 (57.1) | | |
| III | 91 | 52 (65.8) | 39 (42.9) | | |
| **TILs** | | | | 0.977 | 0.336 |
| <10% | 112 | 49 (62.0) | 63 (69.2) | | |
| ≥10% | 58 | 30 (38.0) | 28 (30.8) | | |
| **HER2 status** | | | | 1.159 | 0.351 |
| IHC 0 | 72 | 30 (38.0) | 42 (46.2) | | |
| IHC 1+/2+FISH- | 98 | 49 (62.0) | 49 (53.8) | | |
| **Ki-67 expression** | | | | 0.087 | 1.000 |
| <20% | 5 | 2 (2.5) | 3 (3.3) | | |
| ≥20% | 165 | 77 (97.5) | 88 (96.7) | | |
| **p53 expression** | | | | 4.430 | 0.041 |
| Negative | 66 | 24 (30.4) | 42 (46.2) | | |
| Positive | 104 | 55 (69.6) | 49 (53.8) | | |
| **CK5/6 expression** | | | | 1.483 | 0.264 |
| Negative | 62 | 25 (31.6) | 37 (40.7) | | |
| Positive | 108 | 54 (68.4) | 54 (59.3) | | |
| **EGFR exprssion** | | | | 0.127 | 0.820 |
| Negative | 22 | 11 (13.9) | 11 (12.1) | | |
| Positive | 148 | 68 (86.1) | 80 (87.9) | | |
| **AR expression** | | | | 1.291 | 0.329 |
| Negative | 113 | 56 (70.9) | 57 (62.6) | | |
| Positive | 57 | 23 (29.1) | 34 (37.4) | | |
| **Lymphovascular invasion** | | | | 5.468 | 0.022 |
| No | 157 | 77 (97.5) | 80 (87.9) | | |
| Yes | 13 | 2 (2.5) | 11 (12.1) | | |
| **Breast pCR** | | | | 36.537 | < 0.001 |
| pCR | 48 | 40 (50.6) | 8 (8.8) | | |
| non-pCR | 122 | 39 (49.4) | 83 (91.2) | | |
| **NAT regimen** | | | | 12.390 | 0.002 |
| TA | 124 | 60 (75.9) | 64 (70.3) | | |
| TA→TP | 26 | 5 (6.3) | 21 (23.1) | | |
| TP | 20 | 14 (17.7) | 6 (6.6) | | |
| **NAT cycles** | | | | 7.840 | 0.008 |
| 4 | 30 | 7 (8.9) | 23 (25.3) | | |
| >4 | 140 | 72 (91.1) | 68 (74.7) | | |

Supplementary Table 4 Univariate analysis of ultrasonographic characteristics influencing the achievement of ypN0 status in TNBC in training set

| Ultrasonographic characteristics | Total (n=170) | Training set n (%) | Validation set n (%) | t/χ²/Z | *p* value |
|---|---|---|---|---|---|
| **Maximum tumor diameter (mm)** | | | | | |
| Before NAT | | 34 (11, 131) | 40 (10, 121) | -1.855 | 0.064 |
| After NAT | | 20 (0, 92) | 22 (0, 100) | -3.392 | < 0.001 |
| **Tumor remission rate(%, mean±SD)** | | 42.86±29.64 | 30.86±26.04 | 3.831 | < 0.001 |
| **RECIST** | | | | 18.990 | < 0.001 |
| CR | 10 | 9 (11.4) | 1 (1.1) | | |
| PR | 98 | 53 (67.1) | 45 (49.4) | | |
| SD | 59 | 16 (20.2) | 43 (47.3) | | |
| PD | 3 | 1 (1.3) | 2 (2.2) | | |
| **Peritumoral echogenicity pre-NAT** | | | | 2.993 | 0.109 |
| Enhancement | 148 | 65 (82.3) | 83 (91.2) | | |
| Unremarkable | 22 | 14 (17.7) | 8 (8.8) | | |
| **Peritumoral echogenicity post-NAT** | | | | 8.821 | 0.004 |
| Enhancement | 122 | 48 (60.8) | 74 (81.3) | | |
| Unremarkable | 48 | 31 (39.2) | 17 (18.7) | | |
| **Peritumoral echogenicity changes** | | | | 6.164 | 0.021 |
| Unchanged | 142 | 60 (75.9) | 82 (90.1) | | |
| Changed | 28 | 19 (24.1) | 9 (9.9) | | |
| **Posterior echogenicity pre-NAT** | | | | 2.867 | 0.239 |
| Unremarkable | 56 | 31 (39.2) | 25 (27.5) | | |
| Attenuation | 106 | 44 (55.7) | 62 (68.1) | | |
| Enhancement | 8 | 4 (5.1) | 4 (4.4) | | |
| **Posterior echogenicity post-NAT** | | | | 5.371 | 0.068 |
| Unremarkable | 107 | 57 (72.2) | 50 (54.9) | | |
| Attenuation | 60 | 21 (26.5) | 39 (42.9) | | |
| Enhancement | 3 | 1 (1.3) | 2 (2.2) | | |
| **Posterior echogenicity changes** | | | | 0.895 | 0.405 |
| Unchanged | 118 | 52 (65.8) | 66 (72.5) | | |
| Changed | 52 | 27 (34.2) | 25 (27.5) | | |
| **Tumor CDFI signal pre-NAT** | | | | 2.163 | 0.339 |
| Prominent and hypervascular | 127 | 63 (79.7) | 64 (70.3) | | |
| Dot-linear | 26 | 9 (11.4) | 17 (18.7) | | |
| Not observed | 17 | 7 (8.9) | 10 (11.0) | | |
| **Tumor CDFI signal post-NAT** | | | | 6.017 | 0.049 |
| Prominent and hypervascular | 66 | 24 (30.4) | 42 (46.2) | | |
| Dot-linear | 28 | 12 (15.2) | 16 (17.6) | | |
| Not observed | 76 | 43 (54.4) | 33 (36.2) | | |
| **Tumor CDFI signal changes** | | | | 6.324 | 0.042 |
| Unchanged | 84 | 33 (41.8) | 51 (56.0) | | |
| Reduced | 78 | 44 (55.7) | 34 (37.4) | | |
| Increased | 8 | 2 (2.5) | 6 (6.6) | | |
| **Minimal transverse diameter of node (mm)** | | | | | |
| Pre-NAT | | 10 (3, 40) | 13 (5, 36) | -3.375 | 0.001 |
| Post-NAT | | 5 (0, 14) | 8 (0, 27) | -5.763 | < 0.001 |
| **Lymph node remission rate (%，mean±SD)** | | 50.31±25.54 | 36.43±29.21 | 3.274 | 0.001 |
| **Lymph node CDFI signal pre-NAT** | | | | 2.933 | 0.231 |
| Prominent and hypervascular | 82 | 35 (44.3) | 47 (51.6) | | |
| Dot-linear | 15 | 10 (12.7) | 5 (5.5) | | |
| Not observed | 73 | 34 (43.0) | 39 (42.9) | | |
| **Lymph CDFI signal post-NAT** | | | | 22.532 | < 0.001 |
| Prominent and hypervascular | 48 | 9 (11.4) | 39 (42.9) | | |
| Dot-linear | 18 | 13 (16.5) | 5 (5.5) | | |
| Not observed | 104 | 57 (72.2) | 47 (51.6) | | |
| **Lymph node CDFI signal changes** | | | | 10.372 | 0.006 |
| Unchanged | 118 | 47 (59.5) | 71 (78.0) | | |
| Reduced | 45 | 30 (38.0) | 15 (16.5) | | |
| Increased | 7 | 2 (2.5) | 5 (5.5) | | |
| **Hyperechoic medulla visible post-NAT** | | | | 0.867 | 0.474 |
| No | 162 | 74 (93.7) | 88 (96.7) | | |
| Yes | 8 | 5 (6.3) | 3 (3.3) | | |
